# Simultaneous Determination of Droplet Size, pH Value and Concentration to Evaluate the Aging Behavior of Metalworking Fluids

**DOI:** 10.3390/s21248299

**Published:** 2021-12-11

**Authors:** Patrick Wahrendorff, Mona Stefanakis, Julia C. Steinbach, Dominik Allnoch, Ralf Zuber, Ralf Kapfhammer, Marc Brecht, Andreas Kandelbauer, Karsten Rebner

**Affiliations:** 1 Process Analysis and Technology (PA&T), Reutlingen University, Alteburg Str. 150, 72762 Reutlingen, Germany; p.wahrendorff@t-online.de (P.W.); mona.stefanakis@reutlingen-university.de (M.S.); julia.steinbach@reutlingen-university.de (J.C.S.); marc.brecht@reutlingen-university.de (M.B.); andreas.kandelbauer@reutlingen-university.de (A.K.); 2 Institute of Physical and Theoretical Chemistry, University of Tübingen, Auf der Morgenstelle 18, 72076 Tübingen, Germany; 3 Institute of Inorganic Chemistry, University of Tübingen, Auf der Morgenstelle 18, 72076 Tübingen, Germany; 4 Gigahertz Optik GmbH, 82299 Türkenfeld, Germany; d.allnoch@gigahertz-optik.de (D.A.); r.zuber@gigahertz-optik.de (R.Z.); 5 Festo SE & Co. KG, Ruiter Straße 82, 73734 Esslingen, Germany; ralf.kapfhamer@festo.com; 6 Department of Material Sciences and Process Engineering (MAP), Institute of Wood Technology and Renewable Materials, University of Natural Resources and Life Sciences, Gregor-Mendel-Straße 33, 1180 Vienna, Austria

**Keywords:** metalworking fluid, design of experiments, response surface modelling, partial least squares regression, principal component analysis, dynamic light scattering, effective scattering coefficient, absorption coefficient, service life expansion

## Abstract

Metalworking fluids (MWFs) are widely used to cool and lubricate metal workpieces during processing to reduce heat and friction. Extending a MWF’s service life is of importance from both economical and ecological points of view. Knowledge about the effects of processing conditions on the aging behavior and reliable analytical procedures are required to properly characterize the aging phenomena. While so far no quantitative estimations of ageing effects on MWFs have been described in the literature other than univariate ones based on single parameter measurements, in the present study we present a simple spectroscopy-based set-up for the simultaneous monitoring of three quality parameters of MWF and a mathematical model relating them to the most influential process factors relevant during use. For this purpose, the effects of MWF concentration, pH and nitrite concentration on the droplet size during aging were investigated by means of a response surface modelling approach. Systematically varied model MWF fluids were characterized using simultaneous measurements of absorption coefficients µ_a_ and effective scattering coefficients µ’_s_. Droplet size was determined via dynamic light scattering (DLS) measurements. Droplet size showed non-linear dependence on MWF concentration and pH, but the nitrite concentration had no significant effect. pH and MWF concentration showed a strong synergistic effect, which indicates that MWF aging is a rather complex process. The observed effects were similar for the DLS and the µ’_s_ values, which shows the comparability of the methodologies. The correlations of the methods were R^2^_c_ = 0.928 and R^2^_P_ = 0.927, as calculated by a partial least squares regression (PLS-R) model. Furthermore, using µ_a_, it was possible to generate a predictive PLS-R model for MWF concentration (R^2^_c_ = 0.890, R^2^_P_ = 0.924). Simultaneous determination of the pH based on the µ’_s_ is possible with good accuracy (R²_c_ = 0.803, R²_P_ = 0.732). With prior knowledge of the MWF concentration using the µ_a_-PLS-R model, the predictive capability of the µ’_s_-PLS-R model for pH was refined (10 wt%: R²_c_ = 0.998, R²_p_ = 0.997). This highlights the relevance of the combined measurement of µ_a_ and µ’_s_. Recognizing the synergistic nature of the effects of MWF concentration and pH on the droplet size is an important prerequisite for extending the service life of an MWF in the metalworking industry. The presented method can be applied as an in-process analytical tool that allows one to compensate for ageing effects during use of the MWF by taking appropriate corrective measures, such as pH correction or adjustment of concentration.

## 1. Introduction

Metalworking fluids (MWFs) are widely used in the metalworking industry, where metal workpieces are machined, ground or milled. Their applications range from lubrication to cooling and removal of metal shavings. Due to the widespread and extensive use of cooling lubricants, sustainability issues are of particular importance. While eco-friendly alternatives to conventional cooling lubricants are currently being investigated [1,2,3], the extension of the service lives of MWFs is another important strategy for improving sustainability. According to DIN 51385, lubricants can be classified by their various fields of application and by composition [4]. Around 90% of the MWFs used in the metalworking industry are water-miscible [5], including emulsifiable MWFs. Knowledge of the emulsion’s properties, such as MWF concentration, pH and nitrite concentration, is essential for economic, environmental and safety reasons. The MWF concentration changes due to evaporation, wear and bacterial decomposition, which is accompanied by a decrease in the pH. The nitrite concentration requires monitoring for occupational safety reasons, as reactions with amine-containing components of the MWF can lead to the formation of *N*-nitrosamines. Control and adjustment of these properties allows prolonging the service life of a MWF, reducing costs and ensure safe working conditions [6].

VDI guideline 3397 describes general procedures for maintaining MWFs used in machining and forming processes [7]. Commonly, the concentration is determined refractometrically [6,7] or by titration [8]. While refractometric measurements are susceptible to the effect of tramp oil, titrations require qualified laboratory personnel, sampling and preparation, and are time consuming [8,9].

Previous approaches in the literature have investigated the droplet size, since increasing droplet size leads to decreasing lubricity [10]. The aging of the MWF leads to a higher average droplet size as the size distribution widens [6,11]. Turbidity measurements with [11,12,13] or without machine learning [14,15,16] and dynamic light scattering (DLS) [15,17,18] have been used to determine the droplet size. With electrochemical impedance spectroscopy, the influences of MWF concentration and pH were investigated and evaluated by multivariate data analysis [19].

Optical spectroscopy has proven to be a fast and versatile method for non-invasive measurements in many applications [3,20,21,22,23]. However, strongly light-scattering materials such as emulsions pose experimental problems, since the scattered light interferes with the absorbance signals exploited in transmission spectroscopy [24]. Previous applications of optical spectroscopy for the characterization of MWFs were limited to either the prediction of the concentration [9,25] or the determination of the size of the dispersed droplets [11,26]. MWF concentration is determined and predicted using fluorescence, Raman and infrared spectroscopy-based multivariate calibration models [9] or using a univariate approach applying UV–VIS spectroscopy [25].

To our knowledge, so far, no attempts have been reported of using optical spectroscopy for the simultaneous determination of droplet size, MWF concentration and pH using a single sensor-based system.

In this study, the quality of MWFs was investigated through systematic variations of MWF concentration, pH and nitrite concentration according to a face-centered central composite design (FCD). The effects of these factors on MWF quality, especially the droplet size, were evaluated by DLS analysis and spectroscopic determination of the effective scattering coefficient μ’_s_. The comparability of μ’_s_ and DLS measurements was demonstrated by partial least squares regression (PLS-R). μ’_s_ and the absorption coefficient μ_a_ were directly measured simultaneously by a newly developed instrument (SphereSpectro 150H, Gigahertz Optik GmbH) [24,27]. Since the instrument allows the parallel measurement of μ_a_, the MWF concentration was spectrally checked using a PLS-R model. In addition to the droplet size, the determination of the pH based on the µ’_s_ was also performed.

Here, a novel approach is presented that allows the simultaneous determination of MWF concentration, pH, droplet size and their interrelations based on a response surface model (RSM).

## 2. Materials and Methods

### 2.1. Chemicals

1 mol/L hydrochloric acid, 1 mol/L sodium hydroxide solutions and pH 7 and pH 10 buffer solutions were purchased from Carl Roth GmbH + Co. KG (Karlsruhe, Germany). Sodium nitrite p.a. was obtained from Merck KGaA (Darmstadt, Germany). The MWF concentrate “Curtis HiSpeed 441 Eco” used in this paper was produced by Curtis Systems GmbH (Hochheim, Germany).

### 2.2. Sample Preparation

**Figure 1:**
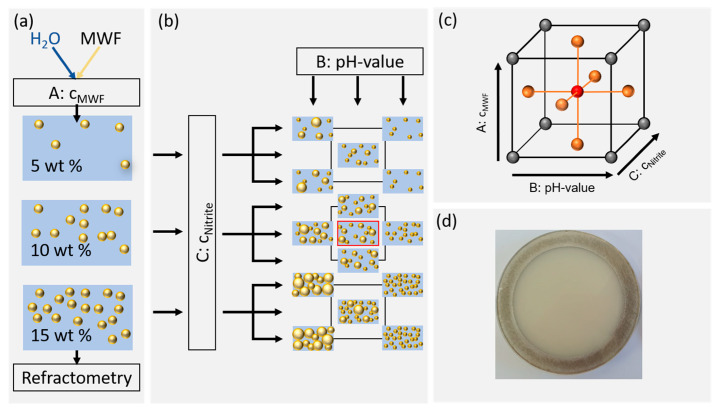
Illustration of sample preparation and the factor level settings according to the response surface methodology. (**a**) Preparation of the desired MWF concentrations and control by refractometry. (**b**) Factor level settings of pH and nitrite concentrations. (**c**) Schematic representation of the examined experimental space. (**d**) Photograph of a typical sample in a quartz cuvette (10 wt% MWF concentration).

The MWF emulsions were prepared using the “Misceo 3” mixing device (Armin Hamma Umwelttechnik, Tuttlingen, Germany). For the mixing process, both the kinematic viscosity of the MWF concentrate at 20 °C and the desired MWF concentration in percent were specified (Figure 1a). The sample composition was systematically varied using a face-centered central composite design (FCD) according to Table 1. Ten liters of emulsion was prepared for each of the three MWF concentrations. The concentrations of the samples were adjusted with deionized water and averaged after twelve consecutive measurements using a digital refractometer (HI96801, Hanna Instruments Deutschland GmbH, Vöhringen, Germany). Sodium nitrite p.a. was weighed (XSE205 DualRange, Mettler Toledo, Gießen, Germany), and then one of the aforementioned emulsions was poured over to a total volume of 250 mL. The pH values of the samples were adjusted with the addition of hydrochloric acid or sodium hydroxide solutions (Figure 1b). All samples were stored at room temperature in amber glass bottles.

### 2.3. Response Surface Model

MWF concentration (factor A), pH (factor B) and nitrite concentration (factor C) were investigated using an FCD (Figure 1c). The Z-average of DLS measurements and µ’_s_ at 650 nm acted as response variables. The factor level limits were determined both by preliminary experiments considering MWF concentrations typically used in the industry [28]. In industrial practice, MWF emulsions outside of pH 8.5–9.5 are usually discarded. The nitrite concentration followed local German safety regulations according to TRGS611 [29]. For MWF emulsions without inhibitor, the upper limit is 20 mg/mL, whereas for MWF emulsions with inhibitor it is 80 mg/mL [30]. For the experimental design, this range was slightly extended to allow accurate interpolations and to allow observing small effects. An overview over the factor settings is given in Table 1.

The planning and analysis of the experiments were performed using Design Expert (version 12, State-Ease Inc., Minneapolis, MN, USA). Only model terms representing statistically significant factor effects and second-order interaction effects were used for model building. A significance level of 5% (*p* < 0.05) was applied.

### 2.4. Determination of the Effective Scattering Coefficient μ’_s_ and the Absorption Coefficient μ_a_

µ_a_ and µ’_s_ were measured using a spectrophotometer (SphereSpectro 150H, Gigahertz Optik GmbH, Türkenfeld, Germany). The instrument was equipped with an integrating sphere (150 mm), a tungsten lamp, a mirror based optical setup for optical beam handling (different beams) and an array-spectroradiometer based detector system (Silicium, Si) and indium gallium arsenide (InGaAs detectors). The spectral range was set to 400–800 nm with a spectral resolution of 1 nm. A maximum integration time of 5 s was selected for the measurements in the absorbance and scattering modes. The samples were filled into a spectrometer-specific quartz cuvette with an optical path length of 3.6 mm (Figure 1d). For the calculation of µ_a_ and µ’_s_ the reflectance and transmittance of a sample are required. Preliminary experiments had shown the suitability of water as an approximation for the MWF emulsions. Therefore, its Sellmeier coefficients were applied [31]. After signal compensation with the calibration standard, the corresponding coefficient value pair was taken from a reference table provided by the instrument manufacturer [32] in which Monte Carlo simulation-based values were collected. The measurements were performed over a period of three days. The time at which a certain measurement was performed was included in the RSM as a block factor in order to account for systematic effects introduced by the actual sequence in measurements. For the determination of factor effects, the values of µ’_s_ recorded at 650 nm were used as the response variable.

### 2.5. Dynamic Light Scattering for Hydrodynamic Radius Determination

The DLS measurements were performed on a Zetasizer-Nano (Malvern Instruments Ltd., Worcestershire, UK) with a wavelength of 633 nm. All samples were diluted by a factor of 1000 with deionized water prior to the measurements, and 10 mm polystyrol cuvettes were used (NeoLab Migge GmbH, Heidelberg, Germany). Three measurements per sample were performed. The intensity weighted mean hydrodynamic size, Z-average was used as the response value for the RSM. It represents a single particle size, which shows a tendency towards higher values as the mean droplet size increases. The refractive indices used for calculation of the Z-average were n*_water_* = 1.333 and n*_MWFconcentrate_* = 1.45.

### 2.6. Multivariate Data Analysis

Multivariate data analysis (MVA) was performed using The Unscrambler X (version 10, Camo Analytics, Oslo, Norway). No additional pre-processing was applied to the data. The principal component analysis (PCA) was calculated with mean centering, an external validation set and singular value decomposition (SVD). The validation set consisted of two additional measurements taken per sample. For model formation of µ’_s_ and µ_a_, a wavelength range of 400–800 nm was used.

Partial least squares regression (PLS-R) models were calculated with mean centering, external validation and a kernel algorithm for the prediction of MWF concentration, using µ_a_ as independent variables. The correlation of Z-average and µ’_s_ was calculated with µ’_s_ as the independent variable and Z-average as the dependent variable. The number of factors for each PLS-R model was optimized for a high coefficient of determination (R^2^) and a low root mean square error of calibration (RMSEC) and prediction (RMSEP). This approach was applied to both the calibration and validation model for each method. A spectral range of 400–800 nm was selected.

The PCA of µ’_s_ was combined with quadratic discriminant analysis (QDA). Two principal components were used to build the PCA–QDA model. The overall accuracy, sensitivity, specificity, false positive rate and precision were calculated based on the confusion matrix terminology [33,34].

## 3. Results and Discussion

### 3.1. Response Surface Model

The effects of the factors MWF concentration, pH and nitrite concentration on the droplet size measured with DLS (Z-average) and µ’_s_ were identified and quantified using response surface methodology. The data are collected in Table 2.

Since the RSM response is restricted to a univariate response input, it was necessary to select a representative µ’_s_ value from the spectrum. µ’_s_ at 650 nm was found to be suitable for evaluating the RSM, as it described the variance within the samples sufficiently. It was located near the center of the visible spectrum, and therefore far enough away from the strongly scattering samples at the lower end and the weakly scattering samples at the upper end of the investigated spectral range.

No significant effect of nitrite content was found for either one of the two models. If one factor turns out to be statistically non-significant in an orthogonal experimental design, such as the FCD for three factors used here, the test becomes equivalent to a complete replication of the experimental design for the two remaining statistically significant factors. This results in experiment STD 5, for example, turning into an indirect (or implicit) replication of experiment STD 1. Thereby, the precision of the experiment was automatically improved. Interestingly, nitrite content does not have any effect at all on MWF quality.

Normal distribution of responses as required for analysis of variance (ANOVA) was achieved through log10 transformation of the raw data. For each response, a model with four significant terms was obtained. The non-significant term A-c_MWF_ for the Z-average model was included to comply with the requirement of preservation of the model hierarchy, which means that all factors that are involved in a second-order interaction must be included in the model regardless whether they are statistically significant by themselves or not [35] (p. 213). The characteristics of both models are shown in Table 3.

The model equation in coded terms gives the effect strength independent of the unit or quantity that is measured. For direct comparison, the model equations for both response surface models are given as Equations (1) and (2).

log10(µ’s) = − 0.3367 + 0.1163 A − 0.4815 B − 0.1550 AB − 0.1195 B²(1)

log10(Z-average) = 1.91 + 0.0021 A − 0.1918 B − 0.0683 AB + 0.0766 A²(2)

Both models showed very good agreement between the predicted and the actual values for the responses (R^2^_µ’s_ = 0.9891 and R^2^_Z__−a__verage_ =0.9557). The values for R^2^_adjusted_ of 0.9858 and 0.9438 for µ’_s_ and z-average, respectively, indicate that no overfitting of the data was present and the number of model terms used in the response surface models was appropriate in relation to the number of experiments performed [20,36]. According to the R^2^_predicted_, both models allowed robust and accurate predictions of the response values within the examined experimental space (R^2^_µ’s_ = 0.9727 and R^2^_Z__−__average_ = 0.9212). The pure error of design was 0.01 for both models, indicating good predictive power of the models within the experimental space studied.

**Figure 2:**
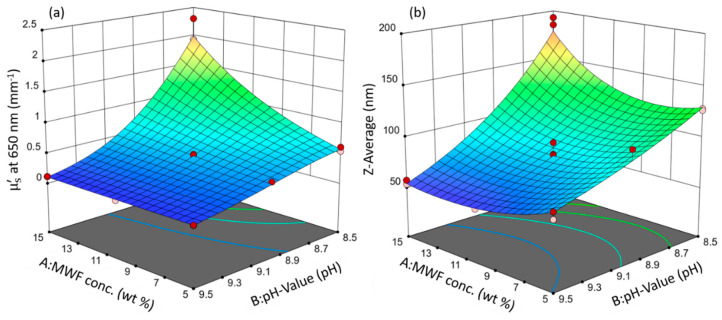
Response surface plots of the models: (**a**) µ’_s_ and (**b**) Z-average. Circles indicate design points. Response values increase from blue to red surface color.

Both models resulted in the same statistically significant factors with the same relative orders of magnitude in effect strength and the same directions (direct or indirect proportionality). These similarities are evident in the three-dimensional response surfaces that were calculated from the model equations, which are depicted in Figure 2.

Since they are involved in a second-order interaction (synergy), neither factor A, the MWF concentration, nor factor B, the pH, can meaningfully be discussed or interpreted independently without simultaneously considering the value of the other factor.

In both models, the pH has an overall decreasing effect on the response value and is the most influential term (highest statistical significance, largest effect term; see Equation (1) and (2)): MWF samples with higher pHs tended to have smaller values for Z-average and µ’_s_. In the case of µ’_s_, this general effect of pH was much less pronounced at lower MWF concentrations (synergy effect; at the lowest concentrations of MWF, there was even no effect of pH at all on µ’_s_). MWF emulsions are designed to be stable in the alkaline range. They are stabilized by an emulsifier, which allows the formation of spherical droplets, and in turn, a minimum contact area between MWF and water [28]. Lowering the pH simulates a form of emulsion aging (biofouling): During regular operation, microorganisms grow on the walls of the storage tanks despite applied biocides. It is known that these microorganisms can metabolize components of the MWF and as a result will lower the pH in the course of their metabolism [37]. With decreasing pH, the negative charges on the emulsifier become neutralized by H^+^. This, in turn, reduces the repulsive electrostatic interaction forces between the droplets and their coagulation is favored. However, with less active emulsifier, still the same quantity of MWF has to be emulsified, leading to larger droplet sizes. This behavior is non-linear for µ’_s_ at 650 nm, as indicated by the negative B² term. The increase in light scattering is more pronounced at lower pHs.

The factor MWF concentration has a minor positive effect on both response variables, but it is of statistical significance only in the model for µ’_s_. It is only included in the Z-average model to preserve model hierarchy.

With increasing MWF concentration, the µ’_s_ value increases. This means that at higher MWF concentrations, generally more droplets form [38]. This increases the number of light scattering events and is consistent with the definition of µ’_s_ as the reciprocal of the average distance light travels between two scattering events [24]. With more droplets present, the mean free path length between them will become increasingly smaller. The Z-average model has a positive non-linear effect term A², reflecting that an increase in droplet size is disproportionally more pronounced as the MWF concentration increases.

**Figure 3:**
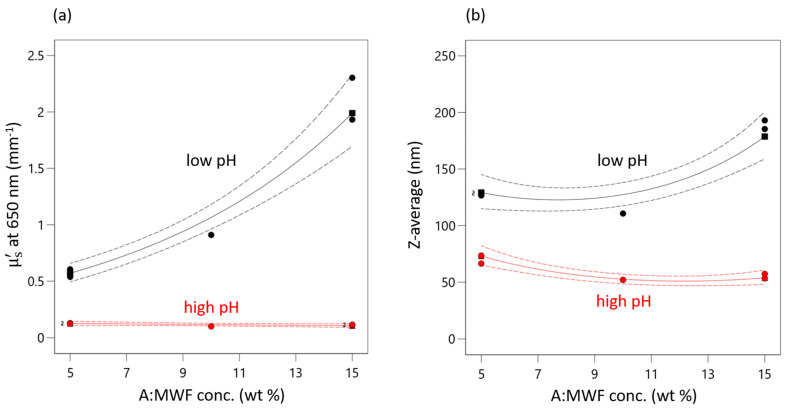
Interaction plot of AB interaction for (**a)** µ’_s_ and (**b**) Z-average. Red triangles indicate responses measured at high pHs. Black squares indicate measured response at low pHs. Green circles indicate measured response at medium pH. Dashed lines indicate 95% confidential intervals.

This positive effect of MWF is only highly pronounced at very low pHs for both responses. pH seems to have a levelling effect on the effect of MWF concentration (as reflected by the second-order interaction effect term). At high pHs, the concentration effect is practically completely absent. This interaction effect is present in both models, has a negative sign and is about one-third the strength of the effect of pH. It is illustrated in the interaction plot for both responses given in Figure 3. As the pH decreases, both response values increase. This increase is more pronounced with increasing MFW concentration.

That at high pHs (red curve) the effect of concentration is negligible can be explained by the pH dependence of the stabilizing effect of the emulsifier, which is obviously highest at pH 9.5. When the emulsion is destabilized by decreasing pH, the droplets start to coagulate and increased scattering is observed. At the lowest pHs (black curve) the emulsifier is destabilized to such an extent that the droplets approach each other and coalesce.

This effect of pH is synergistically reinforced by action of MWK concentration, i.e., an increased number of droplets occurring at higher MWF concentrations, which increases the probability of two droplets colliding and coalescing. At a certain point, the stabilizing effect of the emulsifier is no longer sufficient to prevent coagulation.

This non-linear synergistic behavior is evident from the differently pronounced increases in droplet size at different settings for pH and MWF concentration. For example, at a MWF concentration of 5 wt%, a decrease in pH from 9.5 to 8.5 resulted in an increase in droplet size by 190% (STD 3: 66.4 nm, STD 1: 128.1 nm). In contrast, at a MWF concentration of 15 wt%, the increase (STD 4: 53.3 nm, STD 2: 193.0 nm) was 360% for the same change in pH. Equation (2) can be applied to predict the droplet size for any combination of pH und MWF concentration in the experimental space. Identifying this second-order interaction effect is crucial to prolonging the service lives of MWF emulsions.

The validation of the models’ predictions was performed with two validation points prepared and measured according to Table 2 (entry Val1 and Val2). All predictions were well within ±95% prediction interval (PI, alpha = 0.05); see Table 4. This supports model validity.

The RSM results show the complex influences of the linear, non-linear and interactive effects of MWF concentration and pH on µ’_s_ at 650 nm and Z-average. The similarity of the influences on both response variables demonstrates the comparability of the two measurement methods. MVA, especially PCA and PLS-R, of the µ’_s_ and µ_a_-spectra, was performed to support the results. Using multivariate statistics allows, in contrast to the response surface methodology applied in RSM, the simultaneous consideration of all wavelength-dependent µ’_s_ values and deriving a more comprehensive quantitative regression model.

### 3.2. Multivariate Data Analysis

#### 3.2.1. Correlation of µ’_s_ and Z-Average by PLS-R

PLS-R searches for the optimal correlation between spectral characteristics and an external target value. A PLS-R-model with µ’_s_-spectra as input variables and Z-average as the response variable was used to assess the correlation between the two response variables. Three factors were sufficient for building the model, since they explained 93% of the total variance in the dataset, and additions of more factors did not significantly improve model quality. The PLS-R model of µ’_s_ resulted in high R^2^ and low RMSEC and RMSEP values for the calibration and validation models. Only small differences were observed between the calibration (R²_c_ = 0.938, RMSEC = 10.1 nm) and validation (R²_P_ = 0.927, RMSEP = 10.1 nm) model.

**Figure 4:**
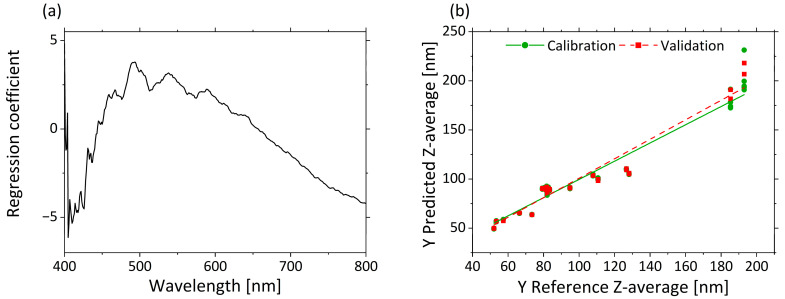
PLS-R with three factors for correlation of µ’_s_ and Z-average. The regression coefficients of the three-factor model are shown in (**a**). Predicted vs. reference of Z-average for calibration (green) and validation (red) are displayed in (**b**).

Figure 4a displays in the regression coefficients for each wavelength in the three–factor model. A negative correlation from 405 to 450 nm and a positive correlation from 450 to 650 nm are clear from the regression coefficients. A negative correlation of the regression coefficients constantly increases again from 660 to 800 nm.

The predicted vs. reference plot in Figure 4b shows the correlation between the Z-average values predicted by the model (y-axis) and the reference values measured with DLS (x-axis). The calibration and validation models are accurate for small droplet sizes. Only for very large droplet sizes (>180 nm) is a comparatively much larger error indicated by the higher deviations of the predicted values. This may result from the different excitation wavelengths used in the two methods, and therefore different accuracies, independently of the droplet size. In summary, a correlation between µ’_s_ and the Z-average was demonstrated. As a result, spectroscopic measurement of µ’_s_ enables the accurate determination and prediction of a quantitative measure for the droplet size.

#### 3.2.2. Effects of pH and MWF Concentration on µ_a_- and µ’_s_-Spectra as Revealed by PCA

**Figure 5:**
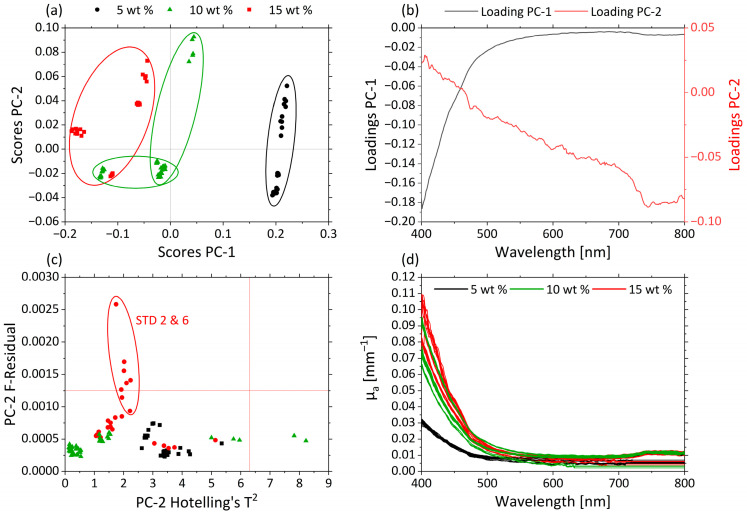
PCA and spectra of µ_a_. (**a**) Scores plot with MWF concentration 5 wt% (black circle), 10 wt% (green triangle) and 15 wt% (red square) for PC-1 against PC-2. (**b**) Corresponding loadings PC-1 (black) and PC-2 (red). (**c**) Influence plot Hotelling’s T^2^ versus F-residuals for PC-2. (**d**) µ’_s_-spectra of MWF concentration 5 wt% (black), 10 wt% (green) and 15 wt% (red).

**Figure 6:**
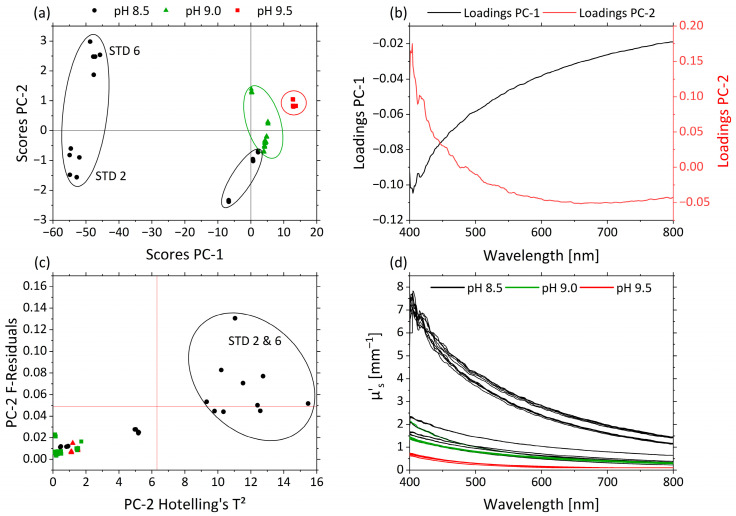
PCA and spectra of µ’_s_. (**a**) Scores plot with pH 8.5 (black circle), pH 9.0 (green triangle) and pH 9.5 (red square) for PC-1 against PC-2. (**b**) Corresponding loadings PC-1 (black) and PC-2 (red). (**c**) Influence plot Hotelling’s T^2^ versus F-residuals for PC-2. (**d**) µ’_s_-spectra of pH 8.5 (black), pH 9.0 (green) and pH 9.5 (red).

Principle component analysis (PCA) structures a dataset by its maximum variance. PCA was applied to the µ_a_- (Figure 5) and µ’_s_-spectra (Figure 6) over the whole range of the visible spectrum from 400 to 800 nm. In Figure 5a the scores values of PC-1 (93.5%) are plotted against those for PC-2 (5.6%) for the µ_a_-spectra.

Both PCs together explain approximately 99% of the total variance within the dataset. The clustering along the PC1 axis reflects differences in MWF concentration of the various samples. The MWF concentrations 5 wt% (black), 10 wt% (green) and 15 wt% (red) are clearly distinguished from each other. Samples containing MWF concentrations of 5 wt% are grouped in the region of positive score values; the 15 wt% are found in the region of negative score values for PC-1; and samples with an intermediate MWF concentration of 10 wt% are found in the central region. Differences in µ_a_ are represented mainly by changes in the spectral region between 400 and 500 nm.

The corresponding loading plots for PC-1 (black) and PC-2 (red) of the µ_a_-based PCA model are displayed in Figure 5b. The highest influence of PC-1 is shown in the spectral range between 400 and 500 nm. For PC-2, the influence of the variables increases continuously with increasing wavelengths.

Samples STD 2 and STD 6 had higher residuals, as can be seen in the influence plot with Hotelling’s T^2^ against F-residuals (Figure 5c). In Figure 5d the µ_a_-spectra are given, and the different samples are colored according to their MWF concentrations: 5 wt% (black), 10 wt% (green) and 15 wt% (red). Higher µ_a_ values occur in the short-wavelength range. With increasing MWF concentration, µ_a_ increases until 500 nm. This can also be seen in the loadings of PC-1. As a consequence, the variations in the MWF concentration dominate the information carried by the µ_a_-spectra. This allowed deriving predictive models for the MWF concentration based on µ_a_.

In Figure 6a the scores of PC-1 (99.6%) against PC-2 (0.3%) are shown for µ’_s_. Both PCs together explain nearly 100% of the total variance within the dataset. The influence of the pH was correlated with PC-1 and PC-2. Samples with different pHs appear as separate clusters along the PC-2 axis. pHs 9.5 (red), 9.0 (green) and 8.5 (black) are separated from one another. pHs 9.5 and 9.0 are grouped above-average on PC-1 and PC-2, respectively. pH 8.5 is divided into three groups based on the effect of STD 2 and STD 6 and is positioned almost completely below-average for PC-1. This is attributed to the strong non-linear behavior of the droplet size and hence µ’_s_ as a function of pH. These experiments were affected by the interaction between pH and MWF concentration, and thus show high variance in the pH 8.5 data, as also indicated by the RSM.

The corresponding loading plots for PC-1 (black) and PC-2 (red) of the µ’_s_-based PCA model are displayed in Figure 6b. The loading values of PC-1 are negative, and the ones < 475 nm of PC-2 are positive. Their trajectories are mirrored with main influences of both PCs in the area <475 nm. The influence plot with Hotelling’s T^2^ against F-residuals shows the strong influences of STD 2 and STD 6 on the model, according to the RSM (Figure 6c). In Figure 6d the µ’_s_-spectra are illustrated and colored according the pHs 8.5 (black), 9.0 (green) and 9.5 (red). Higher µ’_s_ values occur in the short-wavelength range. With decreasing pH, µ’_s_ generally increases, and the decrease in µ’_s_ from the short- to long-wavelength range becomes more pronounced.

STD 2 and STD 6 show high F-residuals and Hotelling’s T² values (Figure 6c) and are obviously clustered in terms of scores, indicating a lack of fit of the PCA model for these samples. STD 2 and STD 6 are crucial for the functionality of the model and were retained. As can be seen in Table 2, both samples had a high MWF concentration and a low pH. The only difference between STD 2 and STD 6 was their nitrite concentration. In RSM, this combination of factor level settings led to especially high values of µ’_s_ and caused the response surface to be non-linear with respect to the factor pH. The protonation of the emulsifier in combination with the high MWF concentration enhanced the coagulation of the droplets.

By increasing the droplet size, the ratio of droplet size and wavelength shifts, leading to increased scattering, especially in the lower wavelength range. This indicates a transition from Rayleigh scattering (*λ << d*) to stronger Mie scattering (*λ ≈ d*), explaining the disproportionate increase in µ’_s_ [39] (136f), [40] (p 361).

While the strong wavelength dependence of Rayleigh scattering (~ *λ^4^*) is reflected by the steep ascent of PC-2 loadings, the flatter profile in the PC-1 loadings corresponds to the less wavelength-dependent Mie scattering. With higher pHs, and thus more stable emulsions and smaller droplets, the ratio of Mie scattering is lower (above-average PC-1 scores), and the ratio of Rayleigh scattering increases (above-average PC-2 scores). In particular, this effect was observed for the increased µ’_s_ at wavelengths >500 nm as an offset in samples STD 2 and STD 6.

From the PCA, it can be deducted that changes in pH are the main cause of variance observed in the µ’_s_-spectra. This allowed deriving a mathematical model that accurately predicts the pH.

#### 3.2.3. Determination of MWF Concentration Based on µ_a_

As the PCA shows a grouping of the µ_a_ scores based on the MWF concentrations, quantitative prediction modelling of MWF concentration was performed using PLS-R. The actual MWF concentrations derived from the settings according to Table 2 were used as responses. Three factors were selected for the model generation. The PLS-R model of µ_a_ is characterized by a high R^2^ (R²_c_ = 0.890, R^2^_p_ = 0.924) and low RMSEC = 1.174 wt%; and RMSEP = 0.975 wt% for the calibration and validation models.

**Figure 7:**
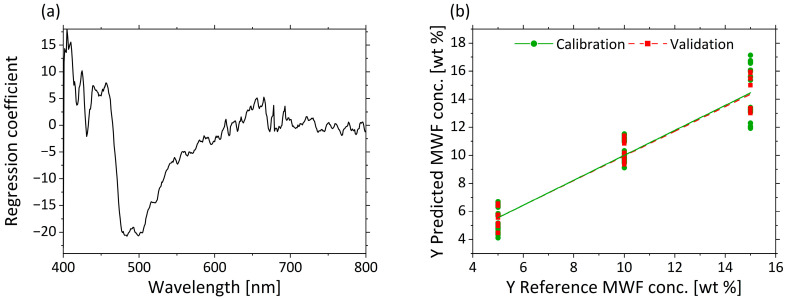
PLS-R with three factors for MWF concentration based on µ_a_-spectra. The regression coefficients of the three-factor model are shown in (**a**). Predicted vs. reference of MWF concentration for calibration (green) and validation (red) are displayed in (**b**).

Figure 7a displays the regression coefficients with three factors. A strong influence from 470 to 550 nm is visible in the regression coefficients. The second important wavelength range is 400–450 nm. The values >600 nm show hardly any influence on the model. The predicted vs. reference plot in Figure 7b shows the correlation between the MWF concentration in wt% predicted by the model (y-axis) and the reference MWF concentration. The calibration and validation models for the low and middle MWF concentration have comparable errors. Despite some larger deviations in the prediction of MWF at high concentration levels (e.g., for 15 wt%, (14.1 ± 1.2) wt%), the spectra of µ_a_ are still considered suitable for the determination of the MWF concentration in a wide concentration range.

#### 3.2.4. Determination of pH Based on µ’_s_

As the PCA shows a clustering of the µ’_s_ scores based on the pH, a prediction of pHs was attempted by PLS-R. Three factors were selected for the model. The interaction between pHs and MWF concentration led to slightly less satisfactory model statistics for pH prediction in the PLS-R model (R²_c_ = 0.803, RMSEC = 0.157, R²_P_ = 0.732, RMSEP = 0.183).

**Figure 8:**
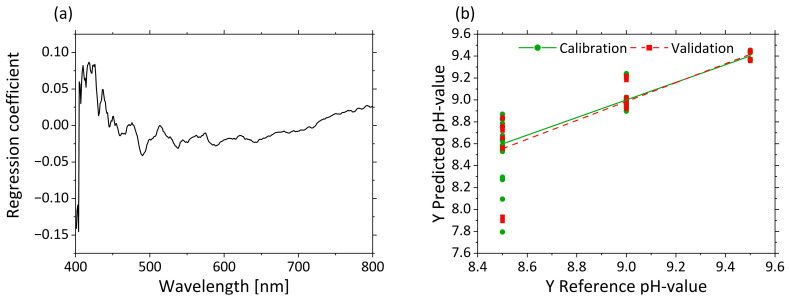
PLS-R with three factors for pHs based on µ’_s_-spectra. The regression coefficients of the three-factor model are shown in (**a**). Predicted vs. reference of pHs for calibration (green) and validation (red) are displayed in (**b**).

Figure 8a displays the regression coefficients with three factors. A strong influence at 405 nm is visible in the regression coefficients. The influence of the regression coefficients increases again from 420 to 500 nm. Above 500 nm, the influence of the variables is constantly at the same level. The predicted vs. reference plot in Figure 8b shows the correlation between the pHs predicted by the model (y-axis) and the reference pHs. The calibration and validation models are accurate for high pHs, but for decreasing pHs, increasing error can be seen by the variance of the predicted values. The predictions of the low pHs led to large deviations (e.g., for pH 8.5, 8.6 ± 0.33).

In order to classify the pHs based on the µ’_s_-PCA model, the scores of PC-1 and PC-2 were used to perform quadratic discriminant analysis (QDA). The confusion matrix resulting from this model is listed in Table 5. The confusion matrix describes the performance of the classification model based on the QDA. The model had an overall accuracy of 99.0%, which means that the model can correctly classify µ’_s_-spectra according to pHs. The highlighted diagonal describes how many spectra were predicted by the model as true. Only one spectrum of a mixture with a pH of 8.5 was predicted falsely to have a pH of 9.0. An average sensitivity of 98.6%, a specificity of 99.3%, a false positive rate of 0.7% and a precision of 99.3% were calculated based on the confusion matrix terminology.

This indicates that the PLS-R cannot linearize the data sufficiently well to account for the interaction effect between pH and MWF concentration.

**Figure A1:**
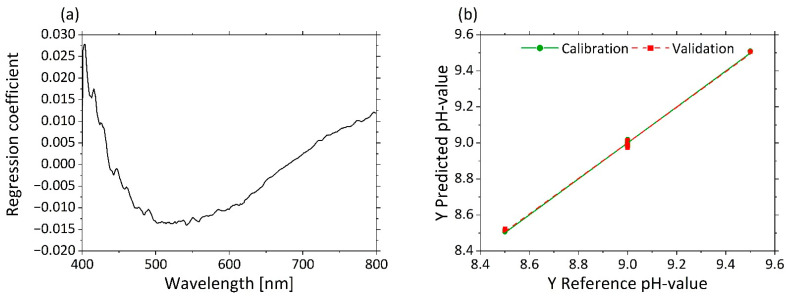
PLS-R with three factors for pHs at 10% MWF concentration values on µ’_s_-spectra. The regression coefficients of the three-factor model are shown in (**a**). Predicted vs. reference of pHs for calibration (green) and validation (red) are displayed in (**b**).

However, it was shown that accurate determination of MWF concentration via µ_a_ is possible. This allowed us to create a successive approach to accurately characterize the MWF emulsion, in which the MWF concentration is first determined by µ_a_ and then the pH is predicted using the PLS-R model of µ’_s_ to the corresponding MWF concentration. When a PLS-R three factor model (Appendix A Figure A1) was implemented using only the data from a defined MWF concentration, 10 wt% in this case, a strong regression model for pH was obtained with R²_c_ = 0.998 and R²_p_ = 0.997, and very minor calibration and validation errors of RMSEC = 0.011 and RMSEP = 0.013, respectively.

## 4. Conclusions

In this work, the effects of the factors MWF concentration, pH and nitrite concentration on the quality decrease of MWF emulsions were studied using droplet size and MWF concentration as quality attributes. To this end, a systematically varied set of MWF emulsions was characterized by spectroscopic methods. The effective scattering coefficients µ’_s_ at 650 nm and Z-average values obtained from DLS measurements were used as response variables. No statistically significant effect was observed for the factor “nitrite concentration.” The MWF concentration and pH showed statistically significant and non-linear effects on droplet size and µ’_s_. In particular, the second-order interaction effect between concentration and pH revealed that aging of MWF emulsions depends in a rather complex (i.e., synergistic and non-linear) way on the two factors. With decreasing pH, the droplet size and µ’_s_ at 650 nm increase, with the effect being stronger at higher MWF concentrations. The correlation of µ’_s_-spectra and Z-average was demonstrated by PLS-R models: R²_P_ = 0.927. This illustrates the suitability of µ’_s_ as an alternative measurement method of droplet size and MWF emulsion aging.

Based on µ’_s_-spectra, QDA classification by pH was possible with 99% overall accuracy. A quantitative PLS-R prediction with an error of 0.18 and R²_P_ = 0.733 was obtained. The simultaneous measurement of µ_a_ allows the prediction of MWF concentration with R²_P_ = 0.924 and an error RMSEP = 0.97 wt%. The presented method of concurrent measurement of µ’_s_ and µ_a_ thus allows the simultaneous determination and quantification of the droplet size, MWF concentration and pH. In combination with the causal model based on the statistically significant factor effect terms, the current condition of a MWF emulsion could, in principle, be monitored in real-time by means of online sensors.

The model-based prediction of the droplet size, and thus the condition of the MWF emulsion for any combination of pH value and MWF concentration, can improve MWF service lifetime in future applications by simultaneous measurement with only one optical sensor. This analytical approach can be used to readjust pH and MWF concentration in order to prolong the useful service lives of MWF emulsions and increase sustainability in the metalworking industry.

## Figures and Tables

**Table 1:** Factor levels of the FCD.

Factor	Name	Unit	−1	0	+1
A	MWF concentration	%	5	10	15
B	pH	pH	8.5	9.0	9.5
C	Nitrite concentration	mg∙L^−1^	0	50	100

**Table 2:** Experiments conducted according to the face-centered central composite design (FCD), sorted by standard order (STD) according to the Yates nomenclature. The order in which the measurements were actually performed is also given. The factor level settings of each experimental run are given along with the corresponding response values.

		Factor Level Settings			Response Values	
STD	Run	A	B	C		
		C_MWF_	pH Value	C_Nitrite_	µ’_s_	Z-Average
		/wt%		/mg∙L^−1^	/mm^−1^	/nm
**1**	**14**	5	8.5	0	0.538	128.1
**2**	**15**	15	8.5	0	2.303	193.0
**3**	**20**	5	9.5	0	0.129	66.4
**4**	**10**	15	9.5	0	0.112	53.3
**5**	**9**	5	8.5	100	0.606	126.6
**6**	**13**	15	8.5	100	1.932	185.3
**7**	**8**	5	9.5	100	0.124	73.5
**8**	**3**	15	9.5	100	0.116	57.3
**9**	**1**	5	9	50	0.393	107.8
**10**	**17**	15	9	50	0.504	82.0
**11**	**7**	10	8.5	50	0.910	110.7
**12**	**2**	10	9.5	50	0.100	52.0
**13**	**11**	10	9	0	0.451	79.3
**14**	**6**	10	9	100	0.434	81.8
**15**	**16**	10	9	50	0.472	83.3
**16**	**19**	10	9	50	0.458	82.7
**17**	**4**	10	9	50	0.489	81.9
**18**	**5**	10	9	50	0.460	80.6
**19**	**18**	10	9	50	0.462	81.4
**20**	**12**	10	9	50	0.472	94.9
**Val1**	**21**	11.2	8.8	0	0.757	85.2
**Val2**	**22**	13.8	8.7	0	1.040	118.4

**Table 3:** Analysis of variance (ANOVA) for responses µ’_s_ and Z-average.

µ‘_s_						Z-Average					
Source	Sum of Squares	df	Mean Square	F-Value	*p*-Value	Source	Sum of Squares	df	Mean Square	F-Value	*p*-Value
**Model**	2.70	4	0.6749	390.28	<0.0001	**Model**	0.43	4	0.1087	80.84	<0.0001
A-c_MWF_	0.13	1	0.13	76.54	<0.0001	A-c_MWF_	0.00	1	0.00	0.03	0.8578
B-pH	2.31	1	2.31	1335.02	<0.0001	B-pH	0.37	1	0.37	273.73	<0.0001
AB	0.19	1	0.19	109.32	<0.0001	AB	0.04	1	0.04	27.76	<0.0001
B²	0.07	1	0.07	40.24	<0.0001	A²	0.03	1	0.03	21.84	0.0003
**Residual**	0.03	15	0.0017	-	-	**Residual**	0.02	15	0.0013	-	-
Lack of fit	0.02	4	0.0050	8.93	0.002	Lack of fit	0.01	4	0.0036	7.03	0.005
Pure error	0.01	11	0.0006	-	-	Pure error	0.01	11	0.0005	-	-
**Cor total**	2.73	19	-	-	-	**Cor total**	0.4549	19	-	-	-

**Table 4:** Actual vs. predicted values (with low and high 95% prediction interval (PI) with alpha = 0.05) and corresponding residuals for the validation points Val1 and Val2.

STD		µ’_s_	Z-Average
**Val1**	Predicted Value (±95%PI)	0.762 (0.601–0.995)	100.3 (82.7–120.9)
	Actual Value	0.757	85.2
	Residual	−0.005	−15.1
**Val2**	Predicted Value (±95%PI)	1.176 (0.919–1.491)	134.8 (110.4–163.5)
	Actual Value	1.040	118.4
	Residual	−0.136	−16.4

**Table 5:** Confusion matrix of quadratic discriminant analysis (QDA).

	Actual	8.5	9.0	9.5
Predicted				
**8.5**		24	0	0
**9.0**		1	50	0
**9.5**		0	0	25

## Data Availability

Data are available upon request from the authors.

## Abbreviations

µ’_s_	effective scattering coefficient
µ_a_	absorption coefficient
*λ*	wavelength
ANOVA	analysis of variance
C_MWF_	metalworking fluid concentration
C_Nitrite_	nitrite concentration
*d*	diameter
DLS	dynamic light scattering
FCD	face-centered central composite design
i. e.	id est
InGaAs	indium gallium arsenide
mm	millimeter
MWF	metallworking fluids
nm	nanometer
n*_MWFconcentrate_*	refractive index of metalworking fluid concentrate
n*_water_*	refractive index of water
PC	principal component
PCA	principal component analysis
PI	prediction interval
PLS-R	partial least squares regression
QDA	quadratic discriminant analysis
R^2^	coefficient of determination
R²_c_	coefficient of determination for calibration
R²_p_	coefficient of determination for prediction
RMSEC	root mean square error of calibration
RMSEP	root mean square error of prediction
RSM	response surface model
s	second
Si	silicium
STD	standard order
SVD	singular value decomposition
Val	validation point
wt%	weight percent

## Appendix A. Determination of pH Based on µ’_s_ −10 wt%

A PLS-R-model with µ’_s_-spectra at 10 wt% MWF concentration as input variables and pHs as response variables was calculated. Three factors were selected for the model generation. The PLS-R model of µ’_s_ is characterized by a high R^2^ (R²_c_ = 0.998, R²_p_ = 0.997) and low RMSEC = 0.011 and RMSEP = 0.013 values for the calibration and validation models.

Appendix AFigure A1 displays in (a) the regression coefficients with the use of three factors. A strong influence from 405 to 450 nm is visible in the regression coefficients. The predicted vs. reference plot in (b) shows the correlation between the pHs predicted by the model (y-axis) and the reference pHs at MWF concentration 10 wt%.
